# Cardiovascular endurance and psychosocial health predict short- and long-term BMI-SDS reduction: results from the CHILT III program

**DOI:** 10.1007/s00431-023-04876-7

**Published:** 2023-03-03

**Authors:** Nina Eisenburger, Nina Ferrari, David Friesen, Fabiola Haas, Marlen Klaudius, Lisa Schmidt, Susanne Vandeven, Christine Joisten

**Affiliations:** Department for Physical Activity in Public Health, Institute of Movement and Neurosciences, German Sport University, Cologne, Germany

**Keywords:** Weight management, Childhood obesity, Psychosocial health, Self-concept, Physical fitness

## Abstract

This longitudinal study analyzes data from the Children’s Health Interventional Trial (CHILT) III, an 11-month juvenile multicomponent weight management program. The objective is to identify predictors of changes in body mass index standard deviation scores (BMI-SDS), so as to further enable the advancement of existing interventions with sustained impact. This study’s sample consists of 237 children and adolescents with obesity (8–17 years, 54% girls) participating in the CHILT III program between 2003 and 2021. Anthropometrics, demographics, relative cardiovascular endurance (W/kg), and psychosocial health (i.e., physical self-concept and self-worth) were assessed at program entry ($t1$), end ($t2$), and one-year follow-up ($t3$; *n* = 83). From $t1$ to $t2$, the mean BMI-SDS was reduced by −0.16 ± 0.26 units (*p* < 0.001). Media use and cardiovascular endurance at baseline and improvements in endurance and self-worth over the course of the program predicted changes in BMI-SDS (adj. *R*^2^ = 0.22, *p* < 0.001). From $t2$ to $t3$, mean BMI-SDS increased ($M = 0.09 \pm 0.29$, *p* = 0.005). Changes in BMI-SDS from $t2$ to $t3$ were associated with parental education, improvements in cardiovascular endurance and physical self-concept, and BMI-SDS, media use, physical self-concept, and endurance level at program end (adj. *R*^2^ = 0.39, *p* < 0.001).

*  Conclusions*: This study highlights the need for comprehensive, sustainable weight management approaches, in order to sustain the initial treatment benefits. In this context, improvements in cardiovascular endurance and psychosocial health could be essential strategies to pursue in practice, as they significantly predicted reductions in BMI-SDS – both pre- to post-intervention and at follow-up.

*Trial registration*: DRKS00026785; date of registration: 13.10.202, retrospectively registered.

**Table:** 

**What is Known:**
• *Childhood obesity is associated with the onset of noncommunicable diseases, many of which are likely to carry into adulthood. Thus, effective weight management strategies for affected children and their families are vital. However, achieving lasting positive health outcomes with multidisciplinary weight management programs remains challenging.*
**What is New:**
• *According to this study, short- and longer-term BMI-SDS reductions are associated to cardiovascular endurance and psychosocial health. These factors should therefore be given even greater consideration in weight management strategies, as they may be important not only in themselves but also for long-term weight loss (maintenance).*

**Supplementary Information:**

The online version contains supplementary material available at 10.1007/s00431-023-04876-7.

## Introduction

Childhood obesity is related to serious short-term and long-term health problems, including physical comorbidities, such as increased risk of cardiovascular disease, type 2 diabetes, cancer, and metabolic syndrome, as well as psychological impairments, including low self-worth, negative self-concept, stigmatization, body dissatisfaction, and depressive symptoms [1–3]. Obesity and its associated physical and psychosocial impairments can develop at a young age and often persist into adulthood [4].

To address this burden, multidisciplinary approaches to weight management are recommended for affected children and adolescents [5]. They aim to achieve weight loss or weight stabilization and lifestyle changes through several components: nutrition counseling, the promotion of physical activity and fitness, behavioral modification, parental and family involvement, reduction of media use and sedentary behavior, and psychological support [6, 7]. Several studies have demonstrated cost-effectiveness [8] and clinically significant impact of these multicomponent approaches on body mass index standard deviation scores (BMI-SDS), physical activity levels, fitness, and psychosocial well-being in children and adolescents from pre- to postintervention measures [9–12]. Findings from a review of 70 weight management programs, with total trial durations of six months to three years, showed a mean BMI-SDS reduction of −0.06 units in 4019 participants aged 6–11 years [13]. Similar effects of multidisciplinary programs on BMI-SDS reduction were reported in a review of adolescents with obesity aged 12–17 years [14].

However, the authors of these reviews found that the quality of evidence was low in terms of internal and external validity and indicated difficulties in finding high-quality evidence of the long-term effects of multidisciplinary obesity treatment. In line with this, Reinehr and colleagues showed in their study, which included more than 21,000 children and adolescents with obesity, that the majority of treatment centers fail to demonstrate the long-term effectiveness of their interventions because of a lack of documentation and a high dropout rate of 92% of the patients at 2-year follow-up [15]. Similarly, Zolotarjova et al. demonstrated that all 16 studies examined in their review reported initial BMI-SDS reductions after multidisciplinary lifestyle interventions for children with obesity, but these changes were either not measured or not sustained at follow-up (three months to three years after the program end) [16]. The authors conclude that the sustainability of BMI-SDS reduction should be of primary importance to ensure that the treatment benefits are maintained over the longer term.

Therefore, to support long-term treatment success, further longitudinal research is needed. In this respect, identifying predictors of BMI-SDS change is of primary importance to uncover risk and protective factors for lasting program impact which can be targeted in multidisciplinary weight management programs [17]. Earlier longitudinal studies have revealed several predictors of childhood weight gain, including sedentary behavior [18], bullying, teasing, and stigmatization [19] and environmental factors such as access to parks [20]. A closer look at factors which influence weigh regain and sustained weight loss (maintenance) after treatment suggests that long-lasting weight control may be predicted by such factors as the degree of overweight at baseline, initial weight loss success, physical activity levels and media use, and psychosocial health [17, 21–24]. Furthermore, there is evidence that male adolescents are more successful at losing weight and maintaining their weight loss than females [16, 25].

However, findings regarding the influence of self-concept and family demographic variables, such as socioeconomic factors, on weight loss after childhood obesity treatment are inconsistent [22, 26, 27]; and many longitudinal studies are limited by the fact that they are often short-term follow-ups immediately after the intervention or only a few months later [10–12, 25]. In addition, a recent meta-analysis indicated a positive association between exercise interventions and body composition in adolescents with obesity; however, the authors raise concerns about the short duration of the trials they synthesized (6–36 weeks) and the fact that only five studies included a measure of cardiovascular fitness, all of which were found to be suboptimal [28].

Therefore, this study aims to contribute to the existing body of research by analyzing longitudinal data from Germany’s Children’s Health Interventional Trial (CHILT) III, an 11-month outpatient multicomponent weight management program for children and adolescents with obesity. The objective of this study is to determine factors associated with short- and long-term BMI-SDS reduction at program completion and one-year follow-up in order to draw conclusions for the optimization of weight management programs in terms of their long-term outcomes.

## Materials and methods

### Intervention description

This study is based on the analysis of data from CHILT III, an outpatient, multicomponent, family-based program conducted at the German Sport University Cologne from 2003 to 2021. The 11-month program targeted children and adolescents aged 8–16 years with obesity (or overweight if displaying cardiovascular risk factors, such as arterial hypertension or hyperlipoproteinemia) and their families [8]. They were referred to the CHILT III program by pediatricians or health insurers. Before the start of the program, each parent was required to sign a participation agreement for the program, in order to be covered by the health insurance company. The agreement stipulated that children and adolescents and their parents must attend more than 80% of the program’s sessions. Child and parent attendance was documented at each session as required by health insurances.

Based on the guidelines of the Working Group on Childhood and Adolescent Obesity of the German Obesity Association [5], CHILT III was built on the pillars of nutrition, physical activity, medical and psychosocial support, and family involvement. The structure of the program, which can be found as Online Resource, required participants to attend two sessions per week. The first weekly session included three aspects: a medical consultation with a physician, during which height and weight were also determined, a 45-min group nutritional or psychological therapy session (alternating, either with a nutritionist or a social pedagogue and/or psychologist), and a 60-min exercise session conducted by sports scientists. The second weekly session was a 90-min physical activity session, resulting in a total of 150 min of physical activity per week. Parents were also measured and weighed weekly and received one nutritional or psychological counseling session per week. Thus, the children were supervised for a total of around four to five hours per week and the parents for two to three hours per week for 40 weeks (no face-to-face sessions were held during vacations, but “homework,” e.g., a footstep challenge, was assigned).

The counseling sessions for both children and parents addressed healthy eating habits, joint cooking events and grocery shopping, the importance of physical activity versus sedentary behaviors, self-esteem and bullying, behavior modification training, and applying these contents to everyday life. Weight progress was discussed, with children alone or together with parents, to meet individual needs and goals. The sports lessons were guided by a varied, playful exercise program, which was intended to arouse the children’s interest in further participation in sports in their lives, including in local clubs and together with their social environment. The focus lay on individual development of motor skills, coordination, endurance, strength, and a positive association with sports and physical activity as well as self-efficacy and injury prevention. Getting to know different movement possibilities, muscle building and learning sport-specific skills and techniques were other relevant contents. The types of sports that were performed ranged from team sports such as soccer, dodgeball, and basketball to weight training, martial arts, and outdoor sports units such as skateboarding or geocaching. Once a month, one of the exercise sessions was held for the whole family to strengthen team and family dynamics.

Since the program’s inception in 2003, some adjustments have been made. From 2003 to 2011, each cohort was divided into two subgroups, with children over 12 separated from younger participants. This division by age was removed in 2012, due to the introduction of the all-day school concept in Germany. In 2012, a new group was added in a more socially deprived area of the city, with similar content, in addition to the group at the German Sport University. In 2020 and 2021, during the coronavirus (COVID-19) pandemic, the program was implemented digitally during the initial lockdown and thereafter under strict hygiene rules (e.g., adapted exercise sessions) [29].

### Study population and sample size

**Fig. 1:**
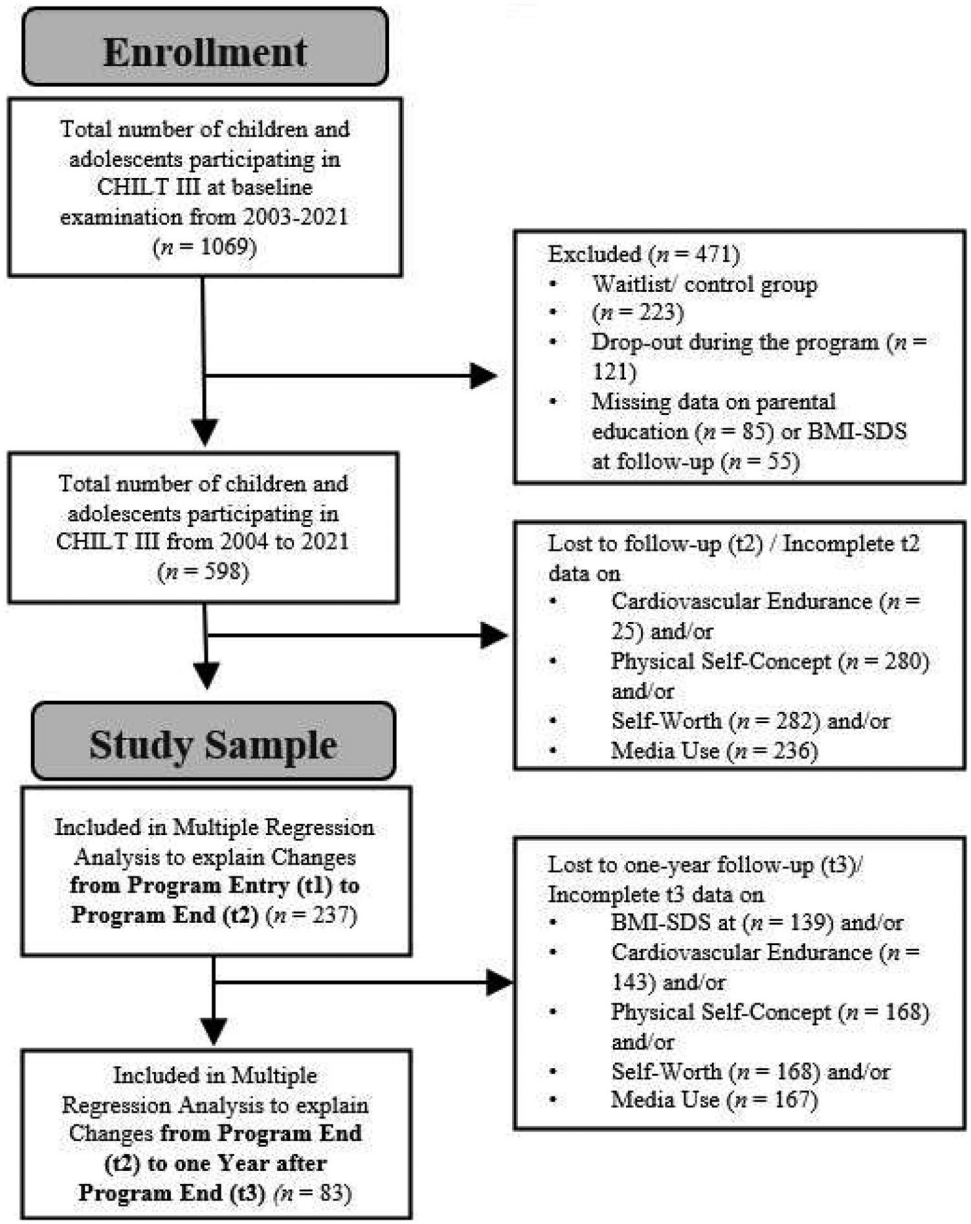
Flowchart of number of participants of this study. CHILT, Children’s Health Interventional Trial; BMI-SDS, body mass index standard deviation scores

Inclusion criteria for participation in CHILT III were, in addition to age and the BMI percentiles mentioned above, e.g., sufficient motivation of children and guardians and active, regular participation, whereas mental or eating disorders or insufficient group ability were considered exclusion criteria for the program. The minimum requirement for each participant to be included in this analysis was participation in the 11-month intervention from beginning to end and complete data on parental education and BMI-SDS at baseline and program end. Further exclusion criteria for this study were missing data at follow-up on BMI-SDS, cardiovascular endurance, physical self-concept, self-worth, or media use (Fig. 1). A final data set of 237 children and adolescents (54% girls) and their parents (*n* = 449: 235 mothers; 214 fathers) remained. An a priori power analysis performed with G*Power 3.1 indicated that at least 83 participants were required for this study in order to perform a multiple linear regression analysis with 15 predictors, a desired large effect size (*f*^2^ = 0.4), and a power of 0.95 at an alpha level of 0.05 [30].

### Anthropometric data assessment

Measurement and weighing of children and adolescents were performed with calibrated standard scales and stadiometer barefoot. The height of children and adolescents was measured barefoot in cm; weight was measured in kg and included clothing, e.g., light sportswear. BMI (weight [kg]/height^2^ [m^2^]) was also assessed. In accordance with the German percentile charts of Kromeyer-Hauschild et al., children and adolescents above the 90th percentile and below or equal to the 97th percentile were considered overweight, and those above the 97th percentile were considered obese [31]. Additionally, sex- and age-specific BMI-SDS were calculated using the following equation: (BMI/*M*(*t*))(*L*(*t*) −1)/(*L*(*t*) × *S*(*t*)), with $M(t)$, $L(t)$, and $S(t)$ reflecting age- and sex-specific parameters for each child [32].

### Demographics and media use

At the beginning and end of the program, as well as one year after program completion, parents completed standardized questionnaires in which they recorded their demographic data and lifestyle habits as well as their children’s. The demographic variables selected for the study were the children’s sex, age, migration background, and parents’ educational background. In cases where the answer to the question about the child’s nationality remained unanswered, the child’s migration background was determined by the language spoken at home (German or non-German) [33]. Parents’ educational backgrounds were divided into three categories. If at least one parent had a high school diploma (in German: *Abitur*), the educational status was classified as high. If at least one parent had a secondary school diploma (in German: *Realschule*), the educational status was classified as medium. Otherwise, i.e., with less than 10 years of schooling (including primary school and *Hauptschule*), the parental educational level was classified as low [34]. In addition, media consumption, a commonly used indicator of sedentary behavior [35], was included. Media use was assessed by asking parents to indicate the total amount of time their child spent per day watching TV, playing on a game console, on the computer, on the internet, listening to music, or using a cell phone. Based on this information, a metric variable for children’s media use in hours per day was coded.

### Cardiovascular endurance

Before the start of the program, cardiovascular endurance was measured in terms of peak mechanical power (PMP [W]) and maximum oxygen consumption (V̇O_2_max [mL/min]) on a bicycle ergometer (Ergoline Ergometrics 900), on which the children and adolescents exercised to exhaustion. After individual adjustment of the bicycle ergometer to the participant, the test began with a load of 25 W and was increased by 25 W every two minutes [10]. Throughout the test, participants were motivated by staff to exert maximum effort. The test was repeated after completion of the 11-month program and one year later. Due to a larger sample size at the program end, peak mechanical power (*n*_*t*2_ = 237) was used instead of V̇O_2_max (*n*_*t*2_ = 229) as an indicator of cardiovascular endurance. The test results were divided by weight (W/kg) to express relative cardiovascular endurance (RCE).

### Physical self-concept and self-worth

For the assessment of physical self-concept and global self-worth, this study used two subscales that resulted from a German version of Harter’s Self-Perception Profile for Children [36] by Wünsche and Schneewind, named FSK-K (Fragebogen zur Erfassung von Selbst-und Kompetenzeinschätzungen bei Kindern) [37]. In the 30-item questionnaire, each item was rated on a scale of 1 to 4 in an alternative statement format, with a positive statement on one side (e.g., “I like my body the way it is”) and a negative statement on the other side (e.g., “I want my body to be different”). The child/adolescent decided which side of the description was kind of true/almost true/really true for him/her, sometimes with parental assistance. The test was conducted at all three measurement time points ($t1, t2, t3$). Results were adjusted to fall within a range of 0–100 and recoded so that high scores indicated high self-concept/self-worth. Cronbach’s $\alpha$ was calculated for reliability analysis [38]. The internal consistencies of the subscales at baseline ($t1$) and follow-up ($t2, t3$) were *α*_*t*1_ = 0.79; *α*_*t*2_ = 0.81; *α*_*t*3_ = 0.82 for physical self-concept (*n*_*t*1_ = 214; *n*_*t*2_ = 102; *n*_*t*3_ = 72) and *α*_*t*1_ = 0.71; *α*_*t*2_ = 0.80; *α*_*t*3_ = 0.81 for self-worth (*n*_*t*1_ = 210; *n*_*t*2_ = 209; *n*_*t*3_ = 74).

### Statistical analysis

Descriptive statistics at baseline for anthropometric and demographic data, media use, cardiovascular endurance, self-concept, and self-worth are reported as mean ± SD for continuous variables and as frequencies and percentages for categorical variables. Mean changes from baseline to program end are represented as $\Delta t1t2$ and changes from program end to one year later are represented as $\Delta t2t3$. Mean differences in boys’ and girls’ baseline characteristics [39] were analyzed using independent two-tailed t-tests in a between-subjects design. Based on a within-subject design, paired two-tailed t-tests were conducted to detect significant differences from $t1$ to $t2$ and from $t2$ to $t3$.

Backward stepwise multiple linear regression analysis with $p \ge 0.1$ for variable removal was performed to examine the predictors of mean changes in $\Delta t1t2$ BMI-SDS and $\Delta t2t3$ BMI-SDS. The predictors included in the regression models are illustrated in Table S2 in the Online Resources, including a dichotomous variable to express the participants’ adolescence stage [40] so as to account for the nonlinearity of the relationship between physical self-concept and age [41].

To identify outliers, leverage values (< 0.2) [42], studentized excluded residuals (< 3 and >  −3) and Cook distances (> 1) were analyzed. No extreme values were found. Homoscedasticity, linearity, and normal distribution are assumed, based on visual inspection of quantile–quantile and scatter plots of the unstandardized predicted values and studentized residuals [43]. It is furthermore presumed that no autocorrelation existed between the residuals, since the Durbin–Watson statistics for all models had values close to 2. No multicollinearity existed between the predictors, as the variance inflation factor values were less than 10 in all regression models [44].

For all statistical analyses, IBM SPSS version 28.0 was used, and significance was set at *p* < 0.05.

## Results

A total of 206 (87.7%) of the participants were classified as obese, and 31 (12.3%) were considered overweight at baseline. Among them, 34 (14.3%) children and adolescents reported a migration background, and 203 were German. In 95 (40.1%) cases, at least one parent had a high-school education, and 101 (42.6%) were categorized as having a medium education level; 41 (17.3%) qualified as having a low education level. Further descriptive sample characteristics at baseline can be found in Table 1.

**Table 1:** Descriptive sample characteristics at baseline ($t1$; *n* = 237)

	**Total**		**Boys** **(**${\varvec{n}}\boldsymbol{ }=\boldsymbol{ }109$**)**		**Girls** **(**${\varvec{n}}\boldsymbol{ }=\boldsymbol{ }128$**)**		${\varvec{p}}$ **value**	**Cohen’s** ${\varvec{d}}$
	Mean	SD	Mean	SD	Mean	SD		
**Age (years)**	12.3	2.1	12.5	1.9	12.2	2.3	0.278	0.06
**Height (m)**	1.58	0.11	1.62	0.12	1.56	0.10	< 0.001	0.55
**Weight (kg)**	75.7	19.4	81.1	21.6	71.2	16.2	< 0.001	0.59
**BMI (kg/m**^**2**^**)**	29.8	4.6	30.5	4.8	29.1	4.3	0.017	0.32
**BMI-SDS**	2.45	0.46	2.47	0.45	2.44	0.48	0.628	0.06
**RCE (W/kg)**	1.7	0.4	1.7	0.4	1.7	0.3	0.188	0.31
**Media use per day (hours)**	3.2	3.1	3.2	3.4	3.2	2.9	0.842	0.03
**Physical self-concept**	56.2	17.2	56.6	16.2	55.9	18.1	0.764	0.04
**Self-worth**	74.5	16.1	74.2	14.5	74.8	1.5	0.378	0.04

*p* value based on independent two-tailed t-tests; significant differences between boys and girls in bold; self-concept and self-worth are based on scores ranging from 0 (lowest) to 100 (highest)

*BMI-SDS* body mass index standard deviation score, *RCE* relative cardiovascular endurance, *SD* standard deviation

From program start to program end, BMI ($t[236] = -4.6$, *p* < 0.001, $d = 0.30$) and BMI-SDS were significantly reduced ($t[236] = -9.7$, *p* < 0.001, $d = 0.63$), corresponding to a reduction in BMI-SDS in 72.2% of participants (Table 2). On average, RCE ($t[236] = 9.2$, *p* < 0.001, $d = 0.60$) and physical self-concept ($t[236] = 2.6$, *p* = 0.005, $d = 0.17$) improved. Self-worth increased marginally, not significantly, from program start to end ($t[236] = 1.5$, *p* = 0.07, $d = 0.10$).

**Table 2:** Mean changes from program start ($t1$) to end ($t2$; *n* = 237)

**Variable**	**Mean (SD)**	$\boldsymbol{\Delta }{\varvec{t}}1{\varvec{t}}2$** (SD)**	${\varvec{T}}$	${\varvec{p}}$ **value**	**Cohen’s** ${\varvec{d}}$
**BMI (kg/m**^**2**^**)**	29.2 (4.7)	−0.5 (1.8)	−4.6	< 0.001	0.30
**BMI-SDS**	2.29 (0.55)	−0.16 (0.26)	−9.7	< 0.001	0.63
**RCE (W/kg)**	1.9 (0.46)	0.2 (0.3)	9.2	< 0.001	0.60
**Media use per day (hours/day)**	3.0 (3.1)	−0.2 (2.5)	−1.1	0.269	0.07
**Physical self-concept**	59.3 (18.3)	3.1 (18.1)	2.6	0.009	0.17
**Self-worth**	76.3 (16.6)	1.7 (18.9)	1.5	0.142	0.10

*p* value based on paired t-test; significant differences between baseline data ($t1$) and data at program end ($t2$) in bold; self-concept and self-worth are based on scores ranging from 0 (lowest) to 100 (highest)

*BMI-SDS* body mass index standard deviation score, *RCE* relative cardiovascular endurance, *SD* standard deviation, *Δt1t2* mean difference in data after 11-month intervention ($t2$) from baseline data ($t1$)

From program end to one year after, the mean BMI ($t[237] = 6.6$, *p* < 0.001, $d = 0.72$) and BMI-SDS increased ($t[236] = 2.9$, *p* = 0.005, $d = 0.33$) in our sample (compare Table 3), corresponding to BMI-SDS reductions in 31.3%, BMI-SDS stabilization in 3.6%, and BMI-SDS increases in 65.1% of participants. The changes in RCE, media use, physical self-concept, and self-worth from $t2$ to $t3$ were positive but not significant (all *p* > 0.05).

**Table 3:** Mean changes from program end ($t2$) to one-year follow-up ($t3$; *n* = 83)

	**Mean (SD)**	$\boldsymbol{\Delta }{\varvec{t}}2{\varvec{t}}3$**(SD)**	${\varvec{T}}$	${\varvec{p}}$ **value**	**Cohen’s** ${\varvec{d}}$
**BMI (kg/m**^**2**^**)**	31.4 (5.0)	1.7 (2.4)	6.6	< 0.001	0.72
**BMI-SDS**	2.47 (0.56)	0.09 (0.29)	2.9	0.005	0.33
**RCE (W/kg)**	1.8 (0.5)	0.0 (0.3)	0.0	0.990	0.00
**Media use per day (hours/day)**	4.2 (4.5)	0.5 (4.5)	0.1	0.323	0.11
**Physical self-concept**	61.4 (17.8)	0.2 (14.7)	0.2	0.836	0.02
**Self-worth**	78.6 (18.4)	2.3 (14.8)	1.4	0.170	0.15

*p* value based on paired t-test; significant differences between program end ($t2$) and one year later ($t3$) in bold; self-concept and self-worth are based on scores ranging from 0 (lowest) to 100 (highest)

*BMI-SDS* body mass index standard deviation score, *RCE* relative cardiovascular endurance, *SD* standard deviation, $\Delta t2t3$ mean difference in data from program end ($t2$) to one year later ($t3$)

Table 4 summarizes the results of a backward multiple linear regression analysis explaining the changes in BMI-SDS. Concerning the reductions in BMI-SDSs from pre- to postintervention measurements, improvements in RCE ($\beta = -0.35$, *p* < 0.001) and self-worth ($\beta = -0.23$, *p* = 0.001), as well as baseline media use ($\beta = 0.14$, *p* = 0.048) and RCE ($\beta = -0.14$, *p* = 0.022), were significantly associated with reductions in BMI-SDS. Together with sex ($\beta = 0.10$, *p* = 0.069), age ($\beta = 0.36$, *p* = 0.001), adolescence stage ($\beta = 0.26$, *p* = 0.009), baseline self-worth ($\beta = -0.14$, *p* = 0.050), and $\Delta t1t2$ media use ($\beta = 0.11$, *p* = 0.087), these determinants explained approximately 22% of the variance in $\Delta t1t2$ BMI-SDS (adjusted *R*^2^ = 0.22, *p* < 0.001).

**Table 4:** Final models from backward stepwise multiple linear regression analysis* explaining changes from program entry and program completion

**Model**	**Final predictors**	${\varvec{\beta}}$	**SE**	${\varvec{p}}$ **value**
$\boldsymbol\Delta\boldsymbol t\mathbf1\boldsymbol t\mathbf2$ **BMI-SDS** **(*n*** **= 237)**	Sex: female^a^	0.10	0.03	0.069
	$t1$ age (years)	0.36	0.01	0.001
	$t1$ adolescent stage: adolescent^b^	0.26	0.05	0.009
	$t1$ RCE (W/kg)	−0.14	0.04	0.022
	$\Delta t1t2$ RCE (W/kg)	−0.35	0.06	< 0.001
	$t1$ self-worth	−0.14	0.0	0.050
	$\Delta t1t2$ self-worth	−0.23	0.0	0.001
	$t1$ media use (hours)	0.14	0.01	0.048
	$\Delta t1t2$ media use (hours)	0.11	0.01	0.087
**Adj. *R***^**2**^** = 0.22, **$\boldsymbol F\mathbf{\left({9,227}\right)}\boldsymbol=\mathbf8\boldsymbol.\mathbf3$**, **$\boldsymbol p\boldsymbol<\mathbf0\boldsymbol.\mathbf{001}$				
$\boldsymbol{\Delta }{\varvec{t}}2{\varvec{t}}3$ **BMI-SDS** **(*n*** **=83)**	$t2$ BMI-SDS	−0.41	0.07	0.003
	Sex: female^a^	−0.22	0.05	0.020
	Parental educational level	0.22	0.04	0.025
	$t2$ RCE (W/kg)	−0.48	0.09	0.002
	$\Delta t2t3$ RCE (W/kg)	−0.60	0.09	< 0.001
	$t2$ physical self-concept	−0.26	0.0	0.014
	$\Delta t2t3$ physical self-concept	0.23	0.0	0.020
	$t2$ media use (hours)	−0.25	0.01	0.007
**Adj. *R***^**2**^** = 0.39, **$\boldsymbol F\mathbf{\left({8,74}\right)}\boldsymbol=\mathbf7\boldsymbol.\mathbf7$**, **$\boldsymbol p\boldsymbol<\mathbf0\boldsymbol.\mathbf{001}$				

$\Delta t1t2$ difference in data after 11-month intervention ($t2$) from baseline data ($t1$), $\Delta t2t3$ difference in data from t2 to one year after the intervention ($t3$), *RCE* relative cardiovascular endurance in W/kg; reference categories: ^a^male, ^b^child (< 12 years)

^*^p ≥ 0.1 for stepwise variable removal; excluded variables in $\Delta t1t2$ BMI-SDS model: $t1$ physical self-concept, $\Delta t1t2$ physical self-concept, $t1$ BMI-SDs, migration background, parental educational level; excluded variables in $\Delta t2t3$ BMI-SDS model: $t2$ age, adolescent stage, $\Delta t2t3$ media use, $\Delta t1t2$ BMI-SDS, $\Delta t2t3$ self-worth, $t1$ self-worth, migration background

The reductions in BMI-SDS from $t2$ to $t3$ were related to sex ($\beta = -0.22$, *p* = 0.020), parental education ($\beta = 0.22$, *p* = 0.025), parallel improvements in RCE ($\beta = -0.60$, *p* < 0.001), and physical self-concept ($\beta = -0.23$, *p* = 0.02), as well as data at program completion on BMI-SDS ($\beta = -0.41$, *p* = 0.003), media use ($\beta = -0.25$, *p* = 0.007), and physical self-concept ($\beta = -0.26$, *p* = 0.014). Approximately 39% of the variance was explained by the remaining predictors in the final $\Delta t2t3$ BMI-SDS model (adjusted *R*^2^ = 0.39, *p* < 0.001). All other confounders showed no significant correlations with the dependent variables in the regression analysis.

## Discussion

Our findings support the short-term effectiveness of multidisciplinary lifestyle interventions for children and adolescents with obesity: During the German 11-month CHILT III program, BMI-SDS was significantly reduced, and cardiovascular endurance and physical self-concept were improved. However, consistent with the literature [16], our results indicate difficulties in maintaining weight loss after completion of the program. After one year, participants’ BMI-SDS increased by a mean of 0.09 units. Nonetheless, among the participating children and adolescents, approximately 35% were able to maintain or reduce their BMI-SDS at follow-up. In this respect, higher levels of cardiovascular endurance and psychosocial health were identified as particularly favorable factors for short- and longer-term BMI-SDS reduction.

Increased cardiovascular endurance has been found to be inversely associated with BMI-SDS and to lead to greater retention of a healthy lifestyle through increased intrinsic motivation and enjoyment of physical activity [7, 28]. However, to the best of our knowledge, this is the first study to confirm the role of cardiovascular endurance among relevant predictors of (long-term) weight loss and to show that both improvements in endurance and baseline endurance are important for BMI-SDS reduction. Additionally, our results highlight the role of psychosocial health in reducing BMI-SDS in the short and long term which has also been suggested in previous studies [21, 22]. For example, Buscemi et al. found that long-term weight maintenance at a 10-year follow-up after a multidisciplinary lifestyle intervention was associated with higher psychological quality of life.

Since cardiovascular endurance and psychosocial health are among the very parameters that may distinguish children who can lose or maintain weight (loss) over the long term from those who cannot, we conclude that they should be specifically promoted. Unlike other unmodifiable factors such as age, sex, or parental education, these parameters provide starting points for intervention. Progressive training of moderate intensity in a playful group setting [5, 11, 28, 45, 46], motivational interviews, realistic goal setting, and self-control training could be central strategies for enhancing physical and psychosocial well-being and achieving the desired long-term outcomes [24, 47].

Furthermore, and notably, although the mean BMI-SDS of participants increased from the end of the CHILT III program to one year later, cardiovascular endurance, physical self-concept, and self-worth remained stable and even showed a marginally positive trend (despite the partial weight regain). We therefore advocate not only considering anthropometric outcomes as criteria for weight management program success but also properly attending to other parameters measuring cardiovascular risk or psychosocial health [24].

In addition, we found a higher BMI-SDS at the program end to be a significant predictor of BMI-SDS reductions from $t2$ to $t3$. On the one hand, this result is promising, because even children with severe obesity can experience weight loss [27]; on the other hand, it suggests that higher BMI-SDS at the end of the program and the associated psychological distress may be motivators for change [26]. In line with this, we hypothesize that higher levels of media use at the end of the program—which showed a positive association with $\Delta t2t3$ BMI-SDS improvements in our study—may increase psychological distress and hence motivate change [48]. Nevertheless, our results admit of some tension because we observed different associations between media use and BMI-SDS reduction from $t1$ to $t2$. Because media use and its value to young people have continued to increase—especially during the COVID-19 pandemic [49]—and because both the risk and the potential of media use in relation to weight management have been recognized [18, 48, 50], further longitudinal research with larger samples is needed to draw therapeutic implications (e.g., toward telehealth strategies).

Lastly, we identified parental education, sex, age, and stage of adolescence as significant predictors in our models. In contrast, initial weight loss did not predict long-term changes in BMI-SDS in this study, though it did in other studies [21, 27]. In alignment with Moens et al., we suspect that additional posttreatment care may bias the predictive potential of initial program effectiveness for long-term outcomes [22]. Since sex differences in weight and weight-related behavior, such as physical activity, physical fitness, and mental health impairments, are well established [25, 39, 50], individualized strategies for boys and girls in weight management programs are essential. Moreover, our results highlight the importance of early intervention and parental involvement, given the influence of familial background and the fact that older children were less successful in terms of BMI-SDS reductions during the program in this study [16, 51].

### Limitations

The extensive data set and standardized testing procedures used are among the strengths of this study, but it has limitations. We attempted to include multiple confounding factors, but due to incomplete data, which in turn resulted in a reduction in sample size, several other factors relevant to obesity could not be included. Such additional influences, e.g., dietary habits, parental BMI, and pathology, which we were unable to account for, could also have influenced BMI-SDSs [21, 22]. In addition, for the parameters studied, insufficient data existed in the control group. Because the participants were a treatment-seeking population, the study furthermore has a selection bias. Some data were self-reported or assessed by parents. Thus, information bias and bias due to social desirability cannot be discounted. A further limitation is the high variability in our sample and the sample size, which was large at program start and program end but significantly smaller at $t3$, one year after program completion.

## Conclusion

This study aimed to contribute to a deeper understanding of the predictors of changes in BMI-SDS, by analyzing longitudinal data of the CHILT III program, a German outpatient weight management program for children and adolescents with obesity. Our results from pre- to postintervention measurements ($\Delta t1t2$) suggest significant associations between BMI-SDS reductions and cardiovascular endurance, global self-worth, age, adolescence status, and media use. One year after the program ended, cardiovascular endurance, physical self-concept, parental education, sex, BMI-SDS at program end, and media use predicted the magnitude of changes in BMI-SDS ($\Delta t2t3$). This study’s findings therewith highlight the importance of promoting psychosocial health, e.g., physical self-concept and self-worth, as well as cardiovascular endurance in particular, for holistic short- and long-term weight management. Unlike the other variables we studied, these parameters can be specifically targeted in interventions and thus may play important roles in further advancing weight management strategies with lasting impacts.

## Supplementary Information

Below is the link to the electronic supplementary material.

Supplementary file1 (PDF 114 KB)

Supplementary file2 (PDF 125 KB)

## Data Availability

The data cannot be shared publicly and will not be held in a public repository, because it is collected from a small group of participants, a vulnerable population of children and adolescents with obesity, which involve sensitive patient information and indirect identifiers that may risk the identification of study participants. Reasonable data requests from researchers who meet the criteria for access to confidential data can be sent to the project director, Prof. Dr. med Dr. Christine Joisten via: German Sport University Cologne, Institute of Movement and Neurosciences, Department for Physical Activity in Public Health, Am Sportpark Muengersdorf 6, 50933 Cologne, c.Joisten@dshskoeln.

## Abbreviations

BMI-SDS: Body mass index standard deviation scores
cm: Centimeters
CHILT: Children’s Health Interventional Trial
COVID-19: Coronavirus disease 2019
kg: Kilogram
*M*: Mean
PMP: Peak mechanical power
RCE: Relative cardiovascular endurance
SD: Standard deviation
SE: Standard error
W: Watt
