# Supplementary material for: Cardiovascular endurance and psychosocial health predict short- and long-term BMI-SDS reduction: results from the CHILT III program
Source: Eur J Pediatr. 2023 Mar 3;182(5):2225–34. doi: 10.1007/s00431-023-04876-7 (PMC9982786; doi:10.1007/s00431-023-04876-7)
Supplement: Supplementary file 1 — Supplementary file1 (PDF 114 KB) [file 431_2023_4876_MOESM1_ESM.pdf]

Cardiovascular Endurance and Psychosocial Health Predict Short- and Long-term BMI-SDS Reduction – Results from the CHILT III Program

European Journal of Pediatrics

Nina Eisenburger,[1]* Nina Ferrari,[1] David Friesen,[1] Fabiola Haas,[1] Marlen Klaudius,[1] Lisa Schmidt,[1] Susanne Vandeven,[1] Christine Joisten[1]

[1]Department for Physical Activity in Public Health, Institute of Movement and Neurosciences, German Sport University, Cologne, Germany

*Corresponding author: ninaeisen@gmail.com

Table S1. CHILT III Program Plan

**CHILT III Program**		
**Nutrition Class by Ecotrophologist**	**Duration/ Frequency**	2x 45 min. per week<br>Alternating: one week for children, the next only for parents, altering with psychological consultation
	**Implementation**	In-person group session
	**Content**	Information on healthy food, clarifying questions, group discussions, joint cooking/ grocery shopping
**Exercise/ Physical Activity by Sport Scientists**	**Duration/ Frequency**	2x per week (1x60 min. and 1x90 min.= 150 min. in total per week)

	**Implementation**	In-person group session, family session once per month
	**Content**	Group and team sports, coordination games, fitness, trust games, self-efficacy
**Psychosocial Counseling by Social Pedagogue and/or Psychologist**	**Duration/ Frequency**	2x 45 min. per week<br>Alternating: one week for children, the next only for parents, altering with nutrition
	**Implementation**	In-person group session, individual or family session by arrangement
	**Content**	Group dynamics, motivation, self-esteem, individual or family consultation
**Office hour/Medical Counseling by Physician**	**Duration/ Frequency**	1 x 15 min. per week (individually), plus 3 x 45 min. per program cycle (group session)
	**Implementation**	Regular in-person individual and family session, irregular group sessions for knowledge transfer
	**Content**	Weighing, co-morbidities, metabolic and pathogenetic aspects of obesity
**Medical Examination by Physician/ sport scientists**	**Duration/ Frequency**	At the beginning and end of the program
	**Implementation**	In-person, individually
	**Content**	Blood pressure measurement, BIA, calipometry, blood sampling, anthropometric data collection, ergometry, exercise testing/ spiro ergometry

CHILT, Children's Health InterventionaL Trial (juvenile weight management program analyzed in this study); BIA, Bioelectrical impedance analysis
