# Supplementary material for: Cardiovascular endurance and psychosocial health predict short- and long-term BMI-SDS reduction: results from the CHILT III program
Source: Eur J Pediatr. 2023 Mar 3;182(5):2225–34. doi: 10.1007/s00431-023-04876-7 (PMC9982786; doi:10.1007/s00431-023-04876-7)
Supplement: Supplementary file 2 — Supplementary file2 (PDF 125 KB) [file 431_2023_4876_MOESM2_ESM.pdf]

Cardiovascular Endurance and Psychosocial Health Predict Short- and Long-term BMI-SDS Reduction – Results from the CHILT III Program

European Journal of Pediatrics

Nina Eisenburger,[1]* Nina Ferrari,[1] David Friesen,[1] Fabiola Haas,[1] Marlen Klaudius,[1] Lisa Schmidt,[1] Susanne Vandeven,[1] Christine Joisten[1]

[1]Department for Physical Activity in Public Health, Institute of Movement and Neurosciences, German Sport University, Cologne, Germany

*Corresponding author: ninaeisen@gmail.com

Table S2. Overview on included Predictors in Multiple Linear Regression Analysis

Variable	Type	Definition
**Sex**	Dichotomous	Participant's sex; 0 = boy, 1 = girl
**Migration Background**	Dichotomous	Participant's nationality; 0 = Non-German, 1 = German [1]
**Parental Education**	Metric	Parental educational level; 1 = low, 2= medium, 3= high [2]
**Data at Baseline (t1) and at Program completion (t2)**		
**t1, t2 Age**	Metric	Participant's age in years
**t1, t2 BMI SDS**	Metric	Participant's body mass index standard deviation score[3]
**t1, t2 RCE**	Metric	Maximum power output in watts related to weight (W/kg)
**t1, t2 Media Use**	Metric	Parent-reported media use (hours/day)

**t1, t2 Adolescence Stage**	Dichotomous	Participant's adolescence stage in years; 0 = child (<12 years), 1 = adolescent (≥ 12 years)[4]
**t1, t2 Physical Self Concept and Self-Worth**	Metric	Participants' psychosocial health [5, 6]
**Mean changes from baseline (t1) to program end (t2) and from program end (t2) to one year later (t3)**		
**Δt1t2 BMI SDS**	Metric	Mean changes in BMI SDS
**Δt1t2, Δt2t3 RCE**	Metric	Mean changes in relative cardiovascular endurance (W/kg)
**Δt1t2, Δt2t3 Media use**	Metric	Mean changes in media use (hours/day)
**Δt1t2, Δt2t3 Physical Self-Concept**	Metric	Mean changes in Physical SC
**Δt1t2, Δt2t3 Self-Worth**	Metric	Mean changes in Self-Worth

BMI SDS, body mass index standard deviation score; RCE, Relative Cardiovascular Endurance; Parental education based on highest school degree; Δt1t2, difference in data after 11-month intervention (t2) from baseline data (t1); Δt2t3, difference in data from program end (t2) to one year later (t3); Physical Self-Concept and Self-Worth are based on scores ranging from 0 (lowest) to 100 (highest); t1 and Δt1t2 data were included in regression analysis explaining effects from program start to end (*n*=237); t2 and Δt2t3 data and Δt1t2 BMI SDS were included in regression analysis explaining effects from program end to one-year follow-up (*n*=83)

References

1. Schenk L, Neuhauser H, Ellert U, Poethko-Müller C, Kleiser C, Mensink G. Kinder- und Jugendgesundheitssurvey (KiGGS 2003-2006): Kinder und Jugendliche mit Migrationshintergrund in Deutschland 2008: Robert Koch-Institut. doi:10.25646/3140.
2. Lange D, Plachta-Danielzik S, Landsberg B, Müller MJ. Soziale Ungleichheit, Migrationshintergrund, Lebenswelten und Ubergewicht bei Kindern und Jugendlichen. Ergebnisse der Kieler Adipositas-Präventionsstudie (KOPS). [Social inequality, migration, and healthy environments as determinants of overweight of children and adolescents. Results of the Kiel Obesity Prevention Study (KOPS)]. Bundesgesundheitsblatt Gesundheitsforschung Gesundheitsschutz. 2010;53:707–15. doi:10.1007/s00103-010-1081-4.

3. Kromeyer-Hauschild K, Wabitsch M, Kunze D, Geller F, Geiß HC, Hesse V, et al. Perzentile für den Body-mass-Index für das Kindes- und Jugendalter unter Heranziehung verschiedener deutscher Stichproben. Monatsschr Kinderheilkd. 2001;149:807–18. doi:10.1007/s001120170107.
4. Mensink G, Bauch A, Vohmann C, Stahl A, Six J, Kohler S, et al. EsKiMo - Das Ernährungsmodul im Kinder- und Jugendgesundheitssurvey (KiGGS). [EsKiMo - the nutrition module in the German Health Interview and Examination Survey for Children and Adolescents (KiGGS)]. Bundesgesundheitsblatt Gesundheitsforschung Gesundheitsschutz. 2007;50:902–8. doi:10.1007/s00103-007-0254-2.
5. Wünsche P, Schneewind KA. Entwicklung eines Fragebogens zur Erfassung von Selbst- und Kompetenzeinschätzungen bei Kindern (FSK-K). Diagnostica. 1989:217–35.
6. Harter S. The perceived competence scale for children. Child Dev. 1982:87–97.
